# A Neural Network Model for Learning 3D Object Representations Through Haptic Exploration

**DOI:** 10.3389/fnbot.2021.639001

**Published:** 2021-03-25

**Authors:** Xiaogang Yan, Steven Mills, Alistair Knott

**Affiliations:** Department of Computer Science, University of Otago, Dunedin, New Zealand

**Keywords:** haptic exploration, articulated agent, constrained sequences, path integration, 3D object representations

## Abstract

Humans initially learn about objects through the sense of touch, in a process called “haptic exploration.” In this paper, we present a neural network model of this learning process. The model implements two key assumptions. The first is that haptic exploration can be thought of as a type of *navigation*, where the exploring hand plays the role of an autonomous agent, and the explored object is this agent's “local environment.” In this scheme, the agent's movements are registered in the coordinate system of the hand, through slip sensors on the palm and fingers. Our second assumption is that the learning process rests heavily on a simple model of *sequence learning*, where frequently-encountered sequences of hand movements are encoded declaratively, as “chunks.” The geometry of the object being explored places *constraints* on possible movement sequences: our proposal is that representations of possible, or frequently-attested sequences implicitly encode the shape of the explored object, along with its haptic affordances. We evaluate our model in two ways. We assess how much information about the hand's actual location is conveyed by its internal representations of movement sequences. We also assess how effective the model's representations are in a reinforcement learning task, where the agent must learn how to reach a given location on an explored object. Both metrics validate the basic claims of the model. We also show that the model learns better if objects are asymmetrical, or contain tactile landmarks, or if the navigating hand is articulated, which further constrains the movement sequences supported by the explored object.

## 1. Introduction

How do we acquire knowledge about the objects we encounter in the world? While vision is probably the dominant source of information for most mature adults, our primary source of information about objects comes from the sense of touch. Touch allows an infant to learn the key constitutive property of external objects: they are *solid*. The concept of solidity is cashed out in the terminology of the motor system: a solid surface is something that our hands (and other motor effectors) cannot penetrate. As John Locke noted in his *Essay concerning human understanding*: “If any one ask me, what this solidity is? I send him to his senses to inform him: let him put a flint or a foot-ball between his hands, and then endeavor to join them, and he will know.” Locke argued that representations of objects derived from touch and the motor system are primary, while those derived from vision are only “secondary”: visual representations of objects only acquire their meaning through *associations* with touch-based representations. This argument is still largely unchallenged, but there are relatively few models of how infants acquire touch-based representations of objects. We know this process involves active *exploration* of objects, because touch only delivers information about an object serially, at the points of contact (Dahiya et al., 2009, 2013). We have a good deal of empirical information about the exploratory procedures through which unsighted agents find out about objects (see Lederman and Klatzky, 1987, and much subsequent work). But the brain mechanisms that control these procedures and learn object representations are still not well-understood.

In this paper, we introduce a new model of haptic exploration, that combines two central ideas. The first idea is that haptic exploration can be usefully understood as a form of *navigation*. The second is that the mechanism controlling haptic exploration can be reduced in large part to a domain-general circuit for learning regularities in sequences. We will introduce these ideas in turn.

### 1.1. Haptic Exploration and Whole-Body Navigation

We know a lot about the brain mechanisms that allow an autonomous agent to navigate in a constrained two-dimensional environment, like a room or a maze (O'Keefe and Nadel, 1978; Jeffery, 2018). The hippocampal and parahippocampal circuits involved in this process are among the best understood of all brain mechanisms (Moser et al., 2015; Danjo et al., 2018; Wood et al., 2018). We hypothesize that the brain circuit that controls haptic exploration of objects has computational similarities with the hippocampal circuit that controls whole-body navigation. In the haptic circuit, the navigating “agent” is the hand, and the “local environment” being explored is a three-dimensional object. In whole-body navigation, the agent receives a stream of sensory information about location and movement in an “agent-centered” coordinate system, and must use this to derive an “environment-centered” representation of places in the environment, stable over the agent's movements. In haptic exploration, the relevant sensory modalities are touch sensations: in particular, “slip” sensations that provide information about the hand's movement, picked up by mechanoreceptors in the hand (see e.g., Johansson and Westling, 1984; Westling and Johansson, 1984; Johansson and Flanagan, 2009), and proprioceptive signals that provide information about the hand's current shape (see e.g., Prendergast et al., 2019). These representations are delivered in the “agent-centered” coordinate system of the navigating hand; the task is again to derive representations of place that are stable over hand movements, in a coordinate system centered on the object being explored. Note that both whole-body navigation and haptic exploration allow an important role for “dead reckoning,” where representations of place are updated from movement signals alone.

Conceiving of the hand as a navigating autonomous agent, receiving movement signals in its own coordinate system, also addresses significant problems with existing models of haptic exploration. In most of these models, the hand's movements are represented in a Cartesian coordinate system centered on a point in the world. [For instance, Jamali et al. (2016) and Martinez-Hernandez et al. (2013) use haptic exploration to create point clouds in a world-centered Cartesian reference frame]. But a biological agent doesn't have access to this Cartesian frame: all its information arrives in an agent-centered frame of reference. Moreover, the agent's ultimate goal is to learn a geometric representation of the object in a frame of reference *centered on that object*, which is invariant over changes in the object's environment-centered pose. Our proposed model uses realistic hand-centered movement information, which is defined in direct relation to the navigated object, so it's invariant to the pose of the object. So it uses biologically realistic inputs, and produces pose-invariant object representations.

Obviously, the brain circuits controlling haptic exploration are separate from the hippocampal circuits controlling whole-body navigation (Johansson and Flanagan, 2009). Haptic exploration involves a circuit linking the somatosensory cortex areas registering proprioceptive and touch signals from the hand to the motor cortex areas generating hand movements, via areas in parietal and premotor cortex (Stoeckel et al., 2003; Johansson and Flanagan, 2009). Roland et al. (1998) reported that the lateral parietal opercular involves in discriminating the roughness of objects, and the anterior part of the intraparietal sulcus significantly activates in discriminating the shape and length of objects. Visuomotor neurons in the area F5 of the premotor cortex are found that they do not only discharge when objects are grasped but also when the presentation of particular objects (Murata et al., 1997). But the fact that there are computational similarities between the tasks to be solved suggests that similar brain mechanisms may be involved in the two circuits. This idea is also supported by certain facts about language—in particular, the fact that spatial prepositions can be applied equally well to 3D objects and to 2D environments. For instance, an object can be “in” a room, but also “in” a cup and “in” a box; a moving object can go “across” or “around” a room, but also “across” or “around” a plate; and an object can be “on” the first floor, but also “on” a desk. The similar phenomenon is not only observed in English but also in other languages, such as Mandarin: *yi tiao yu “zai” he li* (a fish is “in” a river) and *yi tiao yu “zai” yu gang li* (a fish is “in” a fishbowl). This suggests that the coordinate systems associated with objects and navigation environments have something in common.

### 1.2. Haptic Exploration and Sequence Learning

The second key idea in our model is that haptic exploration exploits a general-purpose circuit that learns *regularities in sequences*. Haptic exploration is extended in time, as already noted: as exploration proceeds, the agent receives a sequence of tactile sensations and proprioceptive signals. It is useful to note that this sequence is quite tightly constrained by the geometry of the object being explored. (We will assume that in haptic exploration, the hand must stay in contact with the explored object). For instance, consider a hand exploring a cup. There are several tactile states that can only be obtained when the hand is touching the outer surface of the cup *and also the handle*. If the hand then moves away from the handle, maintaining contact with the cup surface, the original tactile state *followed by* the slip sensations associated with the movement uniquely identify a unique point on the cup's surface, in the coordinate system of the cup. In cases like this, *a sequence of haptic signals* carries implicit information about *the object-centered location of the hand*. As a result, a circuit that learns to represent sequences of haptic signals while an object is explored implicitly learns something about the geometry of the object, in the coordinate system of the object.

The idea just outlined is also applicable in 2D navigation: if the 2D environment places constraints on movement sequences, then particular sequences can also implicitly convey information about allocentric location. Note that the idea provides an alternative way of stating the principle of dead reckoning in 2D environments. But in our model, we state this principle in declarative terms: “a sequence of agent-centered perceptual signals encountered during navigation carries (some) information about the agent's allocentric location.” We choose this declarative format because the concept of “allocentric location” is a more complex one for haptic exploration than for whole-body navigation. For whole-body navigation, the agent's “allocentric location” amounts roughly to a point in a 2D map. But a hand is an articulated object, with many degrees of freedom, and it can contact an object in many places: the “allocentric state” of the hand in relation to an object is a point in a fairly high-dimensional space.

In our model, we don't attempt to produce an *explicit* representation of the hand's actual state in relation to the object. We simply rely on the principle that sequences of sensory signals *provide information about* this actual state. The central component of our model is a network that learns declarative representations of *frequently-encountered* sequences of sensory signals. Networks that do this kind of learning are commonplace in the cognitive system: they basically implement a mechanism called “chunking,” which is also implicated in the learning of motor programmes (Averbeck et al., 2002), of idiomatic phrases in language (Tomasello, 2003), and possibly also of event representations (Kurby and Zacks, 2007), so a model of haptic exploration that relies on chunking is a parsimonious one.

To demonstrate the role that a sequence learner can play in a model of navigation, we use a very simple model of sequence encoding, using a self-organizing map (SOM—see section 2.2 for details). There are many far more sophisticated models of sequence processing—for instance, Hierarchical Hidden Markov Models (Patel et al., 2014), long-short term memory models (Hochreiter and Schmidhuber, 1997; Fleer et al., 2020), or more recent transformer models (Devlin et al., 2018; Liu et al., 2019). All of these perform far better at sequence learning than our SOM model, and could readily be used in place of it. But our point in the current paper is just to demonstrate *in principle* how sequence modeling can contribute to a haptic navigation model. For this purpose, a SOM provides a sufficient proof of concept.

A similar point applies to our model of an articulated hand. Accurate hand modeling is a challenging problem, because the hand consists of many connected parts leading to complex kinematics (Gustus et al., 2012; Chamoret et al., 2013). There are very detailed models of the human hand. Some of these focus on anatomical detail: for instance, Chamoret et al. (2013) builds a model derived from medical image scans. Others focus on realistic deformation: for instance, Joo et al. (2018) models a human hand as a rigged mesh, which behaves realistically when animated by moving the finger joints. Stillfried and van der Smagt (2010) build a model using imaging inputs that also supports realistic movements. But full realism of the hand is not the goal in the current paper: we simply want to show that an articulated hand contributes useful information to the sequence-based model of haptic exploration system we are proposing. The extra information comes from the fact that an articulated hand introduces additional *constraints* to the navigation actions that can be performed, which our model can learn from. (For instance, if the finger and thumb of a human hand are touching two orthogonal surfaces of a cube, each digit independently constrains the movement of the whole hand, and thereby constrains the hand-centered navigation actions that are currently possible). To demonstrate how articulation can improve our model of haptic exploration, we compare an unarticulated hand to the simplest possible articulated hand, with a single degree of freedom.

## 2. Haptic Learning Model

### 2.1. Hand and Object Modeling

**Figure 1:**
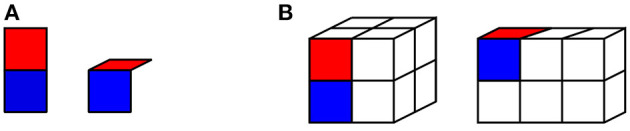
Illustration of the hand used in our model. **(A)** Our hand model, in “straight” position (left) and “bent” position (right). The “palm” is shown in blue; the articulated “finger” in red. **(B)** The objects navigated by the hand in our experiments: a 2 × 2 × 2 cube (left), and a 3 × 2 × 1 cuboid (right). The hand is shown in its “straight” position on the cube, and in its “bent” position on the cuboid.

Our prototype hand has a single degree of freedom, as just noted. It consists of a “palm” and a single “finger” that can be in two positions: “straight” or “bent” at 90 degrees, as shown in Figure 1A. Our hand explores two simple cuboid objects: a 2 × 2 × 2 cube, and a 3 × 2 × 1 cuboid, shown in Figure 1B.

The hand moves in discrete steps on these objects. It has a repertoire of 10 actions, defined in the coordinate system of the hand: translation forward, left, right, or back on the current surface, rotation 90 degrees clockwise or anticlockwise on the current surface, and “bending” and “unbending” movements. Each of these actions has a unique sensory consequence, registered by “slip” sensors on the hand. The physical variable that “slip” sensors measure is the movement of a given surface of the hand in relation to the object being explored (Johansson and Westling, 1984; Westling and Johansson, 1984; Johansson and Flanagan, 2009). At each step, the hand also receives a Boolean representation of the current hand state (straight or bent), and two Boolean signals indicating contact on the finger and the palm.

We must stress the important role of “slip sensors” in registering the hand's movement in our model. As already noted, most models of haptic exploration register the hand's movements in Cartesian coordinates. But a biological agent doesn't have access to this coordinate system: such an agent would have to *compute* Cartesian coordinates through a complex calculation from joint angles. Moreover, the agent doesn't *need* to work in a Cartesian coordinate system: it is much more useful for her to compute her hand's movement *in relation to the object being explored*, because this representation of movement is invariant to changes in the pose of the object. Slip sensors on the hand deliver exactly this object-relative information: it is for this reason that we use these to represent the hand's movements.

Hand actions are constrained by the hand's current configuration and position on the object. The finger can only be bent if it is currently straight, and extended over the edge of a surface. It can only be unbent if it is currently bent. Translation is only allowed if contact with the object is maintained for some part of the hand, and if the configuration of the hand does not prevent movement in the intended direction. These constraints mean that to move from one surface of the cube to another, the hand must move its finger over the edge of one surface, then bend the finger to make contact with the other surface, then straighten the hand.

We also experiment by placing tactile “landmarks” on the navigated object, that uniquely identify a given location. A tactile landmark in our model is a distinctive texture at a given location on the object. With such landmarks we model, for instance, the material difference between the body of a glass bottle and its metal cap, or the open side and the spine of a book. When used, these tactile landmarks are each detected by a dedicated Boolean perceptual signal. Each tactile landmark delivers a unique perceptual signal: no two tactile landmarks deliver the same signal. But note that in our model, tactile landmarks don't provide “ground-truth” information about where the agent is, as they do in some models: they are simply one component of the sequentially structured training data our unsupervised learning model receives. (This usage of the term “landmarks” is also well-attested in the literature on navigation: see for instance Maguire et al., 1999; Chan et al., 2012; Epstein and Vass, 2014). In each test of our experiments, which will be described in section 4, tactile landmarks are randomly distributed on 3D objects. The tactile landmark together with performed actions is input to a recurrent neural network, which will be described in section 2.2.

### 2.2. Sequence Modeling With a Modified Self-Organizing Map

To model frequently-occurring sequences, we use a modified self-organizing map. A self-organizing map (SOM, Kohonen, 1998) is a 2-dimensional array of units, fully connected to an array of inputs by synapses with variable weights. When presented with an input pattern during training, the unit whose synapse weights are closest to this pattern is selected, and its weights are moved incrementally in the direction of the pattern. Units in a Gaussian neighborhood around the winning unit also have their weights adjusted, in proportion to their distance from the winning unit in the two-dimensional grid. During training, SOM units come to represent the most commonly occurring input patterns, and the SOM develops a spatial structure whereby units close together represent similar patterns.

While a regular SOM learns static patterns of inputs, a modified SOM (MSOM, Strickert and Hammer, 2003) takes a sequence of inputs, and its units come to encode commonly encountered sequences in these inputs, through a recurrent connection that maintains a representation of the recent inputs. Our MSOM is trained on the sequence of sensory representations produced when our “hand” agent travels around a cuboid object. Our key hypothesis is that during this training process, units in the MSOM will come to encode the hand's allocentric state on the object. MSOM units learn declarative representations of commonly occurring sequences: because these sequences are constrained by the geometry of the object being explored, we expect MSOM units will end up encoding implicit information about the hand's allocentric state in relation to the object.

Formally, we will define our MSOM as a 2D array of units $M\in R^{n\times n}$. If we present the MSOM with an input pattern $x\left(t\right)\in R^{m}$ at one discrete time instant *t*, with *m* denoting the dimension of *x*(*t*), the activity of a neuron *i* is

(1) $$\begin{array}{r} a_{i}\left(t\right)=\text{exp}\left(-\nu d_{i}\left(t\right)\right), \end{array}$$

where *i* ∈ 1, 2, ⋯, *n*^2^ is the index of a neuron in the MSOM, ν > 0 is a scaling parameter, and *d*_*i*_(*t*) is a distance function. Regarding our model, *x*(*t*) is the input vector, which encodes the hand state, contact information and the action that is performed. *x*(*t*) can also encode tactile landmark perceptual signals when the explored object has tactile landmarks. A neuron in an MSOM has two types of weights: one is a regular weight, which is the same as the weight of a neuron in a regular SOM, and the regular weight is expected to represent *what* is the input pattern; and the other is the context weight, which represents the context information about *when* the represented input pattern occurs. The distance function *d*_*i*_(*t*) shown in Equation (1) is a weighted sum of two parts: the first part is $∥x\left(t\right)-w_{i}\left(t\right){∥}_{2}^{2}$ (this is the regular SOM distance metric), which computes the distance between the input *x*(*t*) and the regular weight *w*_*i*_(*t*) of neuron *i*; and the second part is $∥c\left(t\right)-c_{i}\left(t\right){∥}_{2}^{2}$, which calculates the distance between a context weight *c*(*t*) for the map M and the context weight *c*_*i*_(*t*) of neuron *i*. The distance function *d*_*i*_(*t*) is defined as

(2) $$\begin{array}{r} d_{i}\left(t\right)=\left(1-\zeta \right)∥x\left(t\right)-w_{i}\left(t\right){∥}_{2}^{2}+\zeta ∥c\left(t\right)-c_{i}\left(t\right){∥}_{2}^{2}, \end{array}$$

where ζ ∈ (0, 1) is a weight factor to adjust the effect of such two parts on *d*_*i*_(*t*), and *w*_*i*_(*t*) is the regular weight of neuron *i* in the map that aims to represent some information conveyed in the current input *x*(*t*). The context weight *c*(*t*) for the map M in Equation (2) that represents when the current input *x*(*t*) occurs to the map is computed by

(3) $$\begin{array}{r} c\left(t\right)=\left(1-\iota \right)w_{bmu\left(x\left(t-1\right)\right)}\left(t-1\right)+\iota c_{bmu\left(x\left(t-1\right)\right)}\left(t-1\right), \end{array}$$

where *w*_*bmu*(*x*(*t*−1))_(*t* − 1) and *c*_*bmu*(*x*(*t* − 1))_(*t* − 1) stand for the regular weight and context weight of the neuron in MSOM with the maximal activity (i.e., the best matching unit, BMU) at previous discrete time instant *t* − 1, respectively, and the design parameter ι ∈ (0, 1).

While MSOM units normally have unbounded activity, if we normalize the activity of MSOM units to sum to 1, we can interpret a MSOM pattern as a probability distribution over alternative possible input patterns (and in our case, over alternative possible locations). By norming the activities of all MSOM neurons shown in Equation (1), we have

(4) $$\begin{array}{r} p_{i}\left(t\right)=\frac{a_{i}\left(t\right)}{\sum_{s=1}^{n^{2}}a_{s}\left(t\right)}, \end{array}$$

which denotes the activity probability of neuron *i* for the current input at time instant *t*. With regard to each time instance, all neurons in the MSOM have an activity that is calculated by Equation (1). We term the activity probability of all neurons in the MSOM that is calculated by Equation (4) as *activity pattern*. Based on the distance function shown in Equation (2), the BMU *bmu*(*x*(*t*)) can be determined by

(5) $$\begin{array}{r} bmu\left(x\left(t\right)\right)=\arg\underset{i}{\min}\left(1-\zeta \right)∥x\left(t\right)-w_{i}\left(t\right){∥}_{2}^{2}+\zeta ∥c\left(t\right)-c_{i}\left(t\right){∥}_{2}^{2}. \end{array}$$

During training, the regular weight *w*_*i*_(*t*) is updated as

(6) $$\begin{array}{r} w_{i}\left(t+1\right)=w_{i}\left(t\right)+l\left(t\right)h\left(i,bmu\left(x\left(t\right)\right)\right)\left(t\right)\left(x\left(t\right)-w_{i}\left(t\right)\right), \end{array}$$

and the context weight *c*_*i*_(*t*) is changed as

(7) $$\begin{array}{r} c_{i}\left(t+1\right)=c_{i}\left(t\right)+l\left(t\right)h\left(i,bmu\left(x\left(t\right)\right)\right)\left(t\right)\left(c\left(t\right)-c_{i}\left(t\right)\right), \end{array}$$

where *l*(*t*) and *h*(*i, bmu*(*x*(*t*)))(*t*) are a decreasing learning rate function and neighborhood function respectively, with *bmu*(*x*(*t*)) denoting the index of the neuron in MSOM with the maximal activity for the current input *x*(*t*). At the beginning of training, generally the regular weight *w*_*i*_(0) is initialized to random numbers between 0 and 1, and the context weight *c*_*i*_(0) = 0.

### 2.3. Model Architecture

**Figure 2:**
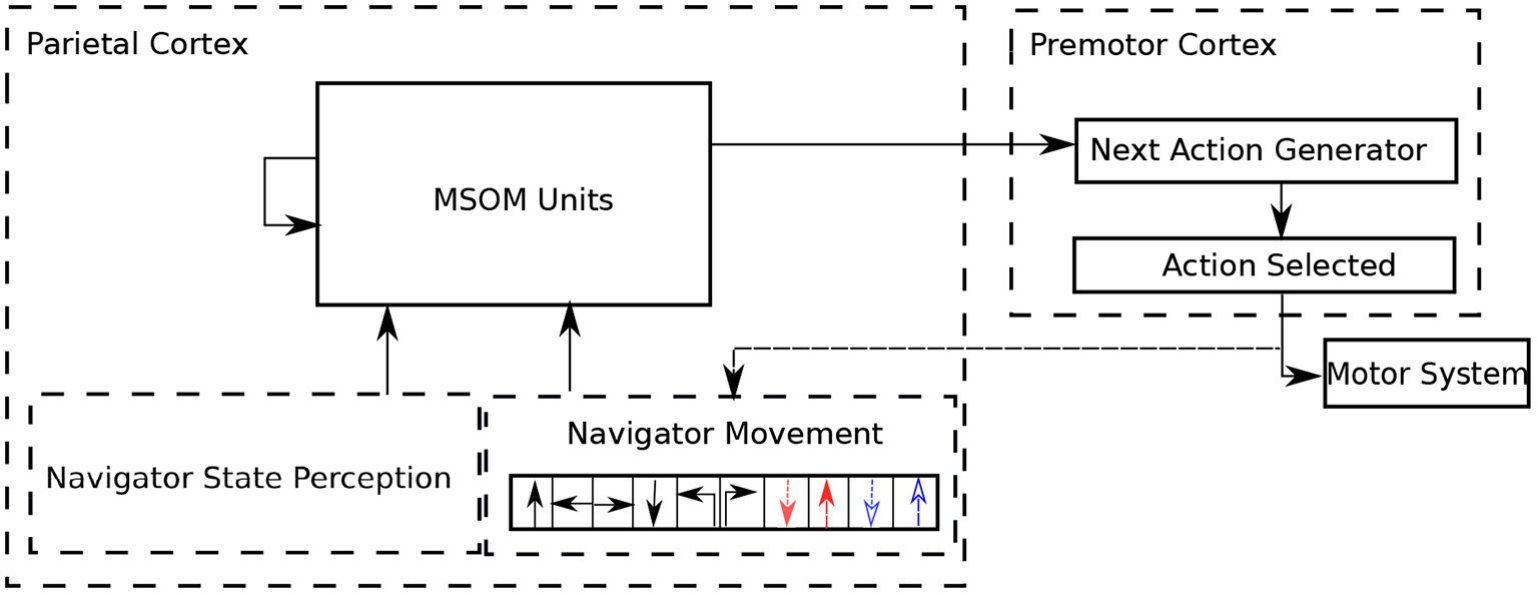
Architecture of the proposed articulated tactile learning model for learning 3D object representations via translative movements (↑: translating forward; ←: translating left; →: translating right and ↓: translating back), orientational movements (↰: orientating 90° counterclockwise and ↱: orientating 90° clockwise), and bending and unbending movements (denoted in dashed colored lines) as well as the navigator/agent state perception, which includes the agent's shape and touching perceptions. MSOM units take the combination of the navigator state perception and the performed movement as their inputs, and then derive an activity pattern calculated in Equation (4). Based on the obtained activity pattern, the next action generator predicts what moves are possible in the current context. A possible action is stochastically selected to be executed. If the action selected is successfully performed, it updates the agent state and the performed action is an input to MSOM units. Otherwise, another action is stochastically selected until an action is successfully executed in the current context.

In the complete haptic exploration architecture, the agent must choose what action to do next, based on its current state. The current state is represented by the current MSOM pattern; based on this pattern, a simple “next action generator” circuit predicts a probability distribution over next moves. This circuit basically learns what moves are *possible* in the current context, and chooses stochastically between those that are deemed possible. If the selected action is possible, it is executed, and the MSOM gets it as an input. The next action circuit also learns that the selected action is possible in the current MSOM state. If the selected action is not possible, the next action circuit learns that the selected action is not possible, and another action is stochastically selected. At the start of exploration, since there is no prior information about the 3D object, all actions predicted by the “next action generator” are with the same probability (i.e., in a uniform distribution). With the exploration proceeding, the MSOM activity pattern gradually associates with object locations, which makes the “next action generator” more accurately predict possible next actions that are allowed by locations on the object (i.e., in a non-uniform distribution). The complete architecture of the proposed articulated tactile learning model is shown in Figure 2.

## 3. Metrics for Evaluating the Model's Ability to Learn Place Representations

Our hypothesis is that the proposed articulated tactile learning model is able to learn something about the agent's location on a simple 3D object. To evaluate this hypothesis, we introduce some metrics for testing what information about the agent's location is conveyed by MSOM activity patterns, after the model has been exposed to a training object for some period of time. All of these metrics evaluate the model from an *external* perspective: we observe the MSOM activity pattern and the agent's actual location over the learning process; then we devise a method to estimate the agent's location on the 3D object based on the MSOM activity pattern; and based on the difference between the estimated location and the actual location of the agent on the 3D object, we evaluate the model's learning performance.

### 3.1. Estimating Agent's Position Based on MSOM Activity Pattern

Assume that a 3D object is partitioned as discrete locations and there are *p* locations on the object. Locations on a 3D object are denoted as *l*_*j*_ with *j* = 1, 2, ⋯, *p*, where *p* stands for the number of locations on the 3D object. For instance, for the 2 × 2 × 2 cube shown in Figure 1B, there are 24 discrete locations on the cube (that is, *p* = 24), and every location on the cube has a unique index among *l*_1_, *l*_2_, ⋯, *l*_24_. We also assume that the MSOM has *n*^2^ units, as defined in Equation (1), and the unit *i* is denoted as *u*_*i*_ with *i* = 1, 2, ⋯, *n*^2^.

Recall that in Equation (1), when an MSOM receives an input, each unit in the MSOM has an activity. Then, we normalize activities of all units in the MSOM to estimate the activity probability of each unit (i.e., the activity pattern), which is computed in Equation (4). We also identify the most active unit: the “best matching unit,” using Equation (5). At each time during the learning process, the agent is in one location *l*_*j*_ and there is a corresponding MSOM activity pattern. We denote the activity probability of the unit *u*_*i*_ as *p*_*u*_*i*__. As the model trains, we use counters in a *hit map* to record the corresponding relationship between the MSOM winner and the agent's actual location. In the hit map, there are counters *c*_(*i*; *j*)_ that record how many times the MSOM winner was unit *u*_*i*_ and the agent's actual location was *l*_*j*_ over a learning period.

For each exploration step, we observe the MSOM winner *u*_*i*_ and the agent's actual location *l*_*j*_; then, we increase the corresponding counter *c*_(*i*; *j*)_ in the hit map by 1. At any given time in training, we can use the hit map to estimate what information about location is conveyed by each MSOM unit. We do this by estimating a conditional probability distribution over locations for each MSOM unit. Given an MSOM activity pattern with the winner being *u*_*i*_, the probability of the agent being in the location *l*_*o*_ is

(8) $$\begin{array}{r} p\left(l_{o}|u_{i}\right)=\frac{c_{\left(i;o\right)}}{\sum_{j=1}^{j=p}c_{\left(i;j\right)}}. \end{array}$$

Thus, the total probability of the agent being in location *l*_*o*_ is

(9) $$\begin{array}{r} p\left(l_{o}\right)=\overset{i=n^{2}}{\underset{i=1}{\sum }}p\left(l_{o}|u_{i}\right)p_{u_{i}}. \end{array}$$

We then rank the locations on the 3D object in order of the corresponding probabilities, as calculated in Equation (9).

### 3.2. Reconstruction Accuracy

The first metric is *P*_max_, which is defined as

(10) $$\begin{array}{r} P_{\max}=\frac{T\left(\alpha =\beta \right)}{\phi } \end{array}$$

where ϕ denotes the size of a sliding window (the sliding window stands for a fixed-length duration that moves one step for every one time instant along the time direction; and we observe criteria of the proposed model over sliding windows), *T*(·) denotes how many times the given event happened in that window, α denotes the actual agent's position, β denotes the most probable position of the agent from MSOM perspective (i.e., the position with highest reconstruction probability calculated in Equation 9). That is to say, *P*_max_ computes how many times the agent's actual location is the location reconstructed with the highest probability over a sliding window. This can be generalized to count how many times the true position is in the top *h* predictions, which gives *P*_max_ for *h* = 1. These results are very similar for a range of values of *h*, so we omit them in this paper.

### 3.3. Geodesic Distance

Reconstruction accuracy metrics like *P*_max_ don't give a quantitative indication of how close an incorrect estimate is to the true location. For this purpose, we use a metric *D*_geodesic_, which reports the sum of geodesic distances between the agent's actual position and each location on the 3D object weighted by the corresponding reconstruction probability calculated in Equation (9). Let *g*(*l*_*o*_, α) denote the geodesic distance between position *l*_*o*_ on the object and the actual agent's position α, which is computed from Dijkstra's algorithm (Goldberg and Tarjan, 1996). Then, we define

(11) $$\begin{array}{r} D_{\text{geodesic}}=\overset{p}{\underset{o=1}{\sum }}g\left(l_{o},\alpha \right)p\left(l_{o}\right) \end{array}$$

where *p* denotes the number of positions on the 3D object as assumed in section 3.1 and *p*(*l*_*o*_) denotes the reconstruction probability for position *l*_*o*_ calculated in Equation (9). The higher value of the reconstruction accuracy metrics suggest better representation ability of the proposed model. In contrast, a smaller value of *D*_geodesic_ indicates the model is better at learning representations of 3D objects.

## 4. Results

### 4.1. Simulation Setup

The 3D objects tested in the proposed model are cuboids, constructed from discrete grids. We used two objects: a 2 × 2 × 2 cube and a 3 × 2 × 1 cuboid. We expect the proposed model to be able to learn more about location on the cuboid than on the cube: the asymmetry of the cuboid provides extra information about which face it is on, which is missing in the fully symmetric cube. The MSOM used in the proposed model is a 10 × 10 map, with the design parameter ζ = 0.4 in Equation (2) and ι = 0.5 in Equation (3). The next action generator in the proposed model is implemented as a 100 × 100 × 10 feedforward neural network.

### 4.2. Effects of Tactile Landmarks and Articulation

In earlier work with a simple unarticulated agent (Yan et al., 2018a,b), we showed that an MSOM model can learn some knowledge about 3D objects through the agent's tactile exploration. We also showed that tactile landmarks on the explored object help the MSOM model encode locations (the more tactile landmarks, the greater accuracy). In this paper, we extend these results to the articulated agent.

We train the proposed articulated tactile learning model to learn about a 2 × 2 × 2 cube with different numbers of tactile landmarks. In the proposed model, each tactile landmark is unique, and thus provides an unambiguous cue to a particular object location. We let the model explore and represent the cube in 30 tests, each using a unique random seed and in each test, tactile landmarks are randomly distributed on the cube. That means the agent explores the cube in different trajectories and thus guarantees that our observations are not due to a specific choice of trajectory.

**Figure 3:**
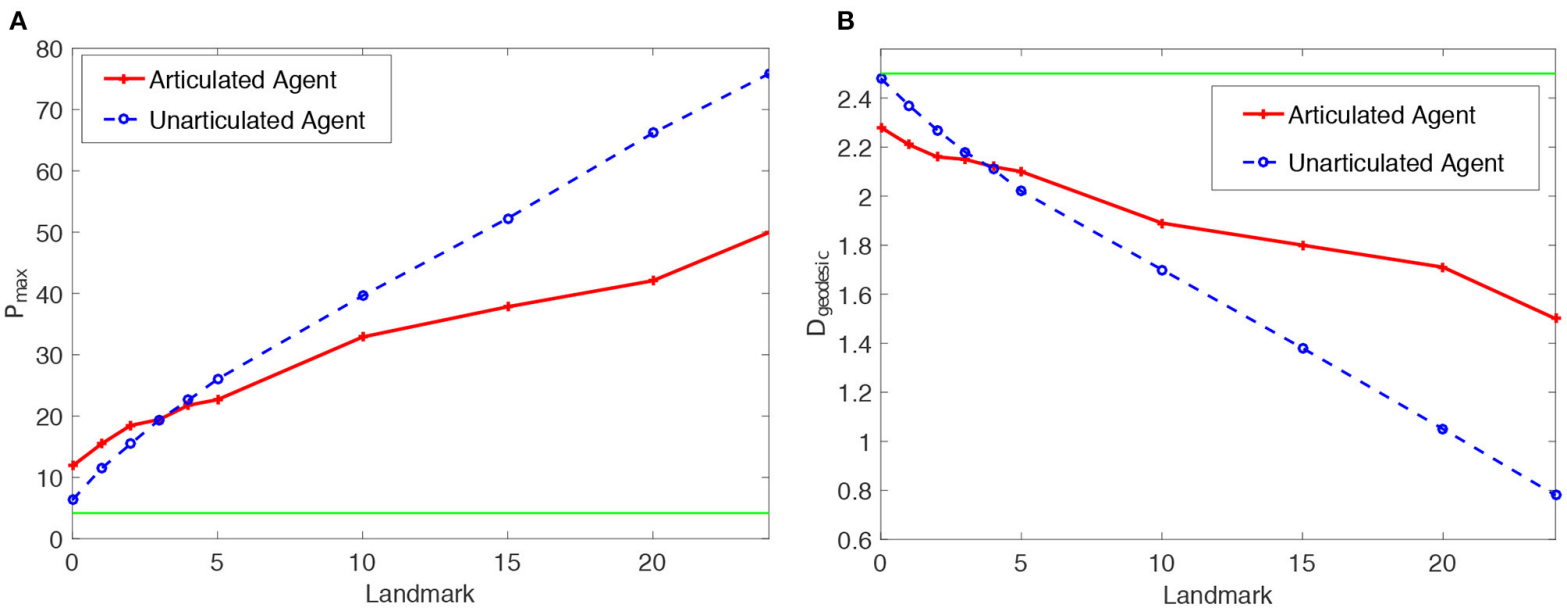
Statistics means of the reconstruction accuracy *P*_max_ and the geodesic distance *D*_geodesic_ for the proposed articulated tactile learning model and the unarticulated model when representing a 2 × 2 × 2 cube. **(A)** *P*_max_ for the models, where the green line stands for the theoretical value of *P*_max_ by random guess. **(B)** *D*_geodesic_ for the models, where the green line stands for the theoretical value of *D*_geodesic_ by random guess.

Results of the proposed articulated tactile learning model on the cube are illustrated in Figure 3. For comparison, in the figure, we also show results of the model with an unarticulated agent (Yan et al., 2018a) and the corresponding theoretical values of evaluation metrics by random guess on the cube. As we can see from Figure 3A:

- Both *P*_max_ of the articulated tactile learning model and the model with an unarticulated agent are greater than the theoretical value of *P*_max_ by random guess, no matter whether the cube is with tactile landmarks or without any tactile landmarks (i.e., the number of tactile landmark being 0). This shows that the models acquire some knowledge about the cube no matter whether there are tactile landmarks or not, and demonstrates the feasibility of using a sequence learner to learn representations of 3D objects through tactile exploration. Therefore, the effectiveness of the proposed model is demonstrated.
- As the number of tactile landmarks on the cube increases, the criterion *P*_max_ rises. This shows the positive effect of tactile landmarks on the proposed model in learning 3D object representations.
- In comparison with the model with an unarticulated agent in Yan et al. (2018a), the proposed articulated tactile learning model is more accurate in learning the representation of the cube, when there are a small number (less than five) of tactile landmarks. For instance, the articulated tactile learning model is superior to the model with an unarticulated agent when the cube is without any tactile landmarks. This shows the configuration information afforded by an articulated agent helps to acquire structure-knowledge of 3D objects. We also observe that the model with an unarticulated agent gains more benefits from tactile landmarks in learning about the cube than the articulated tactile learning model, when the cube is with a large number of tactile landmarks (such as all grids on 3D objects are with tactile landmarks). This is because the articulated agent is more complicated than the unarticulated agent, while in easy cases (e.g., all girds on 3D objects are with tactile landmarks), it is less efficient than the unarticulated one. Note that to make the comparison fair, the MSOMs in the proposed model and the model with an unarticulated agent have the same number of neurons. However, for the articulated agent, there is more perceptual information to be processed: namely the configuration of the agent, and an additional dimension of touch sensation. We believe that when the object has many tactile landmarks, this additional information may overload the capacity of the MSOM. This capacity is not very high: for instance, Vesanto and Alhoniemi (2000) and Tian et al. (2014) argue that the optimal number of neurons in a SOM approximates $5\sqrt{q}$, where *q* denotes the number of input patterns to be learned. (Our future work with higher-capacity models, discussed in section 6.1, may be able to test this hypothesis).

Figure 3B also demonstrates the above conclusions: tactile landmarks and the configuration information afforded by an articulated agent help the model to learn about the structure information of 3D objects.

### 4.3. Effect of Object Asymmetries

In our unarticulated model (Yan et al., 2018a), we showed that asymmetry of the explored object is also an important cue: the model is more accurate on the cuboid than the cube. In this paper, we extend these results to the articulated agent.

Similar to the experiment conducted in section 4.2, we train the proposed model to learn about a 3 × 2 × 1 cuboid with different numbers of tactile landmarks. For a certain number of tactile landmarks, we let the model explore and represent the cuboid in 30 tests. In each test, the random seed is different and tactile landmarks are randomly distributed on the cuboid.

**Figure 4:**
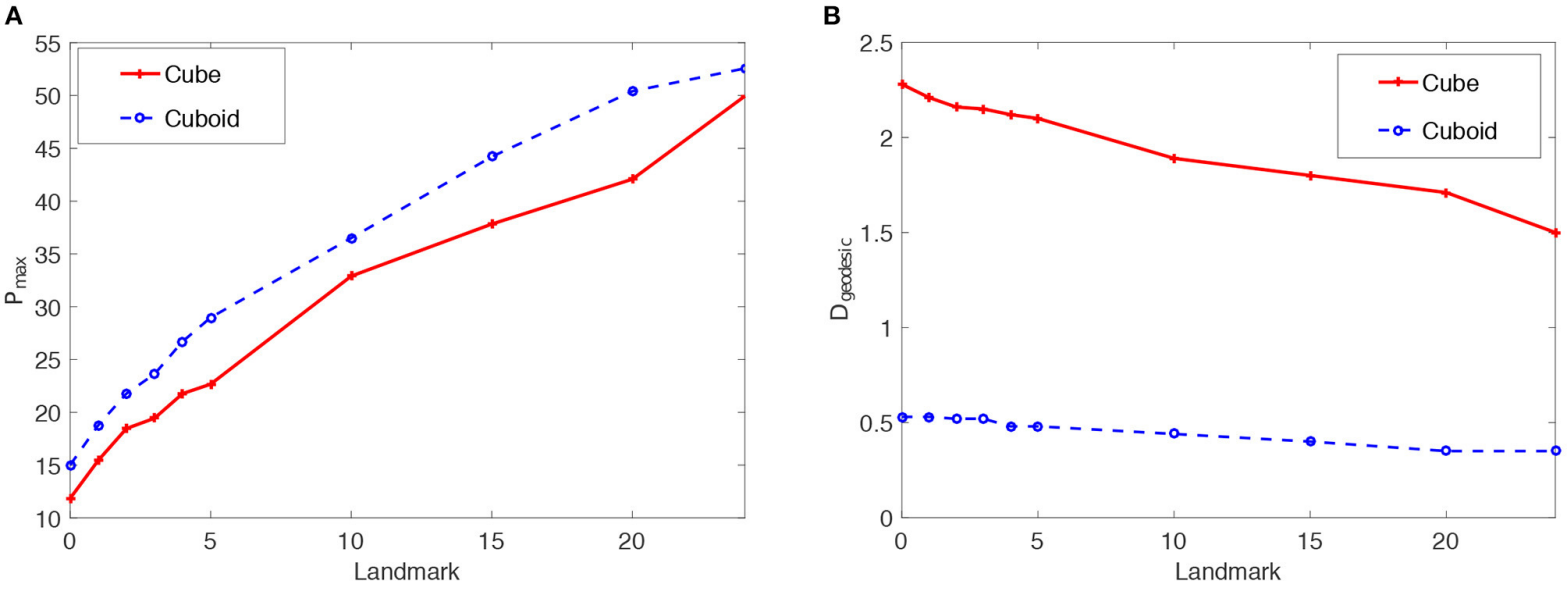
Statistics means of the reconstruction accuracy *P*_max_ and the geodesic distance *D*_geodesic_ for the proposed articulated tactile learning model and the unarticulated model when representing a 2 × 2 × 2 cube and 3 × 2 × 1 cuboid. **(A)** *P*_max_ for the models when representing such two 3D objects. **(B)** *D*_geodesic_ for the models when representing such two 3D objects.

Results of the articulated tactile learning model on the cuboid are shown in Figure 4. Figure 4A shows two results:

- The model has a higher reconstruction accuracy *P*_max_ in learning about the cuboid than the cube. This demonstrates that object asymmetries help the model to learn about structure-knowledge of 3D objects.
- With more tactile landmarks on the cuboid, the model learns a more accurate model of the cuboid. This also shows the positive effect of tactile landmarks on the model's learning performance.

Figure 4B also verifies that object asymmetries and tactile landmarks help the proposed model to acquire structure-knowledge of 3D objects.

## 5. Articulated Goal-Oriented Learning Model

### 5.1. Model Architecture

In section 2.3, we developed a model that learns 3D object representations through tactile exploration by an articulated agent. In section 4, we showed that the proposed MSOM-based model of haptic exploration can learn some information about the navigating agent's location on the exploring object. However, we demonstrated this from a perspective *external* to the agent, with metrics that made reference to the ‘actual’ position of the hand on the object. This is well and good—but we also need an ‘internal’ evaluation, that checks whether the location information expressed in the MSOM is usable *by the agent*, for its own purposes. A reinforcement learning (RL) model is an obvious choice here. If an MSOM activity pattern has some association with the agent's actual allocentric location on the explored object, then this pattern should be able to be used as a proxy for this actual location in a RL scheme. Therefore, in this section, we propose an articulated goal-oriented learning model to explore the hypothesis that the MSOM activity pattern could be used for the agent to reach a goal on a 3D object in the RL domain.

**Figure 5:**
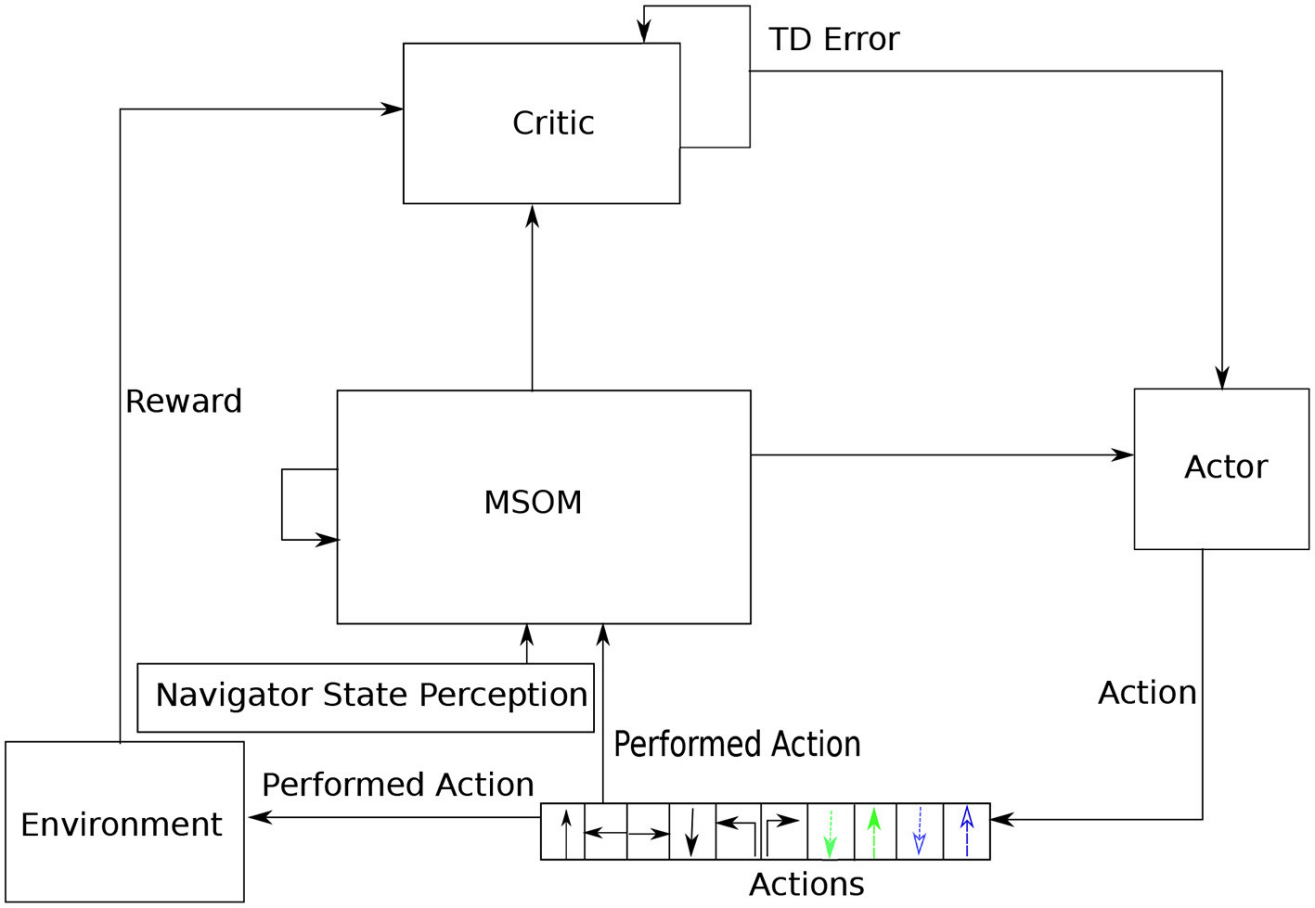
Architecture of the proposed articulated goal-oriented learning model, where ↑ denotes translating forward directly, ← denotes translating left directly, → denotes translating right directly and ↓ denotes translating back directly. Orientating movements are represented as ↰: orientating 90° counter-clockwise and ↱: orientating 90° clockwise. The dashed colored lines stand for bending and unbending movements. First, the MSOM learns stable representations of object locations using the model presented in section 2.3. Then, based on the learned representations in MSOM, an actor critic is employed to achieve the reinforcement learning. When the agent is in one context, MSOM derives an activity pattern. The critic computes the temporal difference (TD) error δ (line 16 in Algorithm 1) based on the current activity pattern and the received reward from the environment (i.e., the 3D object being explored). This TD error is used for updating both the critic (line 17 in Algorithm 1) and the actor (line 18 in Algorithm 1). The actor predicts an action for the agent to perform for reaching a goal on the 3D object.

The architecture of the proposed articulated goal-oriented learning model is shown in Figure 5. The RL algorithm we employ in the proposed model to solve a goal-oriented navigation problem is a temporal difference (TD) RL system (Sutton and Barto, 2018). A classic RL system has two components: an “actor,” that takes a representation of the current state and decides on the next action to perform, and a “critic,” that evaluates the current state in relation to its proximity to reward. The system learns to take actions that achieve good rewards. A TD system can learn to take actions that achieve rewards that are distant in the future, rather than just at the next step. It does this by computing the difference between the critic's prediction about the reward at the next step, and the actual reward at the next step: the so-called “TD error.” We use a TD system, because in our model the reward is only obtained when the agent reaches the specified goal location, so the agent must learn to achieve a temporally distant reward.

In a regular RL system, the actor and critic both take as input the *actual state* of the agent. In the current context, this would be the agent's *actual location* on the cube. But the agent doesn't have direct access to this location: the whole point of our MSOM model is to deliver an approximation of this location, from information the agent does have access to. In our updated model (see Figure 5), the actor and the critic both take input from the MSOM's current activity pattern, which “stands in” for a representation of the agent's actual location on the object. Other than this change, we implement a classical RL system: the critic learns a value function of MSOM activity patterns, which are representations of object locations, and the actor learns to produce actions that optimize expected reward in the near future (specifically, the sum of temporally discounted rewards, as in Sutton and Barto, 2018). We denote the “estimated” state information supplied by the MSOM representation at time instant *t* as *s*_r_(*t*). And we denote the agent's *actual* state on the physical object at time *t* as *s*_p_(*t*).

The training of the proposed goal-oriented learning model that is shown in Figure 5 consists of two stages, as illustrated in Algorithm 1. The first stage (line 2) is to allow the MSOM model to explore the object, and learn its own representations of object locations, using the learning model presented in section 2.3. The second stage (lines 4—21) is a reinforcement learning algorithm, that uses these learned representations of locations as a proxy for the agent's actual location on the object. Between these stages (line 3), we freeze all the MSOM model's weights, so reinforcement learning operates on stable representations.

**Algorithm 1:**
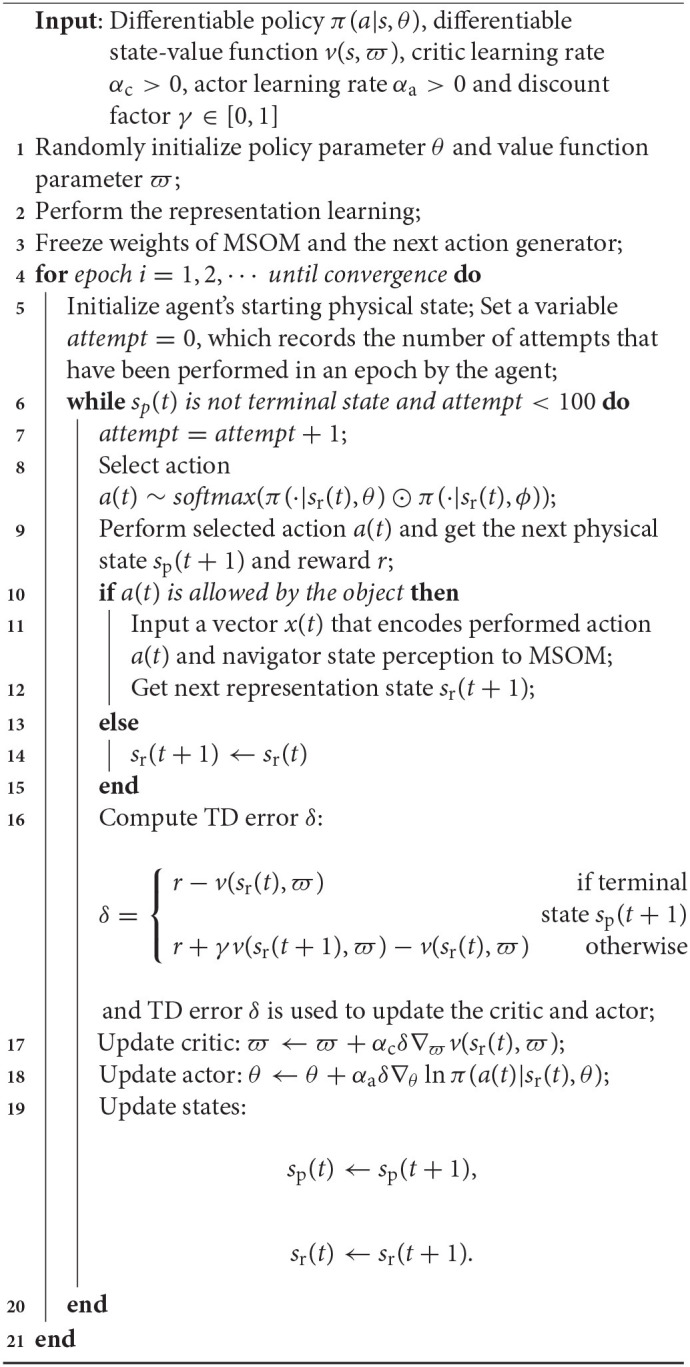
Articulated goal-oriented learning model.

In the reinforcement learning model, the agent selects an action *a*(*t*) from the distribution *a*(*t*) ~ softmax(π(·|*s*_r_(*t*), θ) ⊙ π(·|*s*_r_(*t*), ϕ)), where π(·|·) denotes a policy, θ and ϕ denotes the policy parameter (i.e., weights) of the actor and next action generator, respectively, and ⊙ denotes the element-wise multiplication operation. The knowledge learned in the next action generator helps the agent explore 3D objects more efficiently during reinforcement learning, as the trained generator already knows how to produce successful actions.

### 5.2. Simulation Setup

All parameters involved in the representation learning are the same as those used in previous experiments of the articulated tactile learning model. For instance, the MSOM is implemented as a 10 × 10 map, and the next action generator is implemented as a 100 × 100 × 10 feedforward neural network.

The actor in this goal-oriented learning model is implemented as a 100 × 100 × 10 feedforward neural network and the critic is implemented as a 100 × 100 × 1 feedforward neural network. The learning rate of the actor α_a_ = 0.00001 and the learning rate of the critic α_c_ = 0.0001. During the goal-oriented learning procedure, for each epoch, if the agent does not find the goal within 100 attempts, which includes the successful and unsuccessful attempts, the epoch is automatically finished. On the 2 × 2 × 2 cube, the goal position is opposite to the starting position of the agent (i.e., on the opposite surface) along the diagonal direction. Specifically, the starting position of the agent is set as [surface = 1, x = 1, y = 1], which stands for the top-left position on the cube in Figure 1B that the articulated ‘finger’ is in, and the goal position is set as [surface = 3, x = 1, y = 2], which is opposite to the starting position along the diagonal direction. In the experiment, the reward function is set as,

(12) $$\begin{array}{r} r\left(s_{\text{p}}\left(t\right)\right)=\{\begin{array}{cc} 1, & \text{if }s_{\text{p}}\left(t\right)\text{is a terminal state},\\ -1, & \text{otherwise}. \end{array} \end{array}$$

### 5.3. Results

When studying the performance of the articulated goal-oriented learning model, we run it on the 2 × 2 × 2 cube in 30 tests. Each test has a different random seed. Each test includes 5 × 10^4^ training epochs, with the first 50 epochs for unsupervised representation learning and the rest for the reinforcement learning.

To investigate the performance of the proposed articulated goal-oriented learning model, we compare it with a baseline, in which the same articulated agent performs a random walk on the 2 × 2 × 2 cube. For the random walk condition, we include a simple analog of learning: we assume that in every test, the agent can remember the shortest trajectory that it has experienced. For one epoch in every test, if the current exploration trajectory is longer than its remembered shortest trajectory, we let the remembered shortest trajectory be its current exploration trajectory. Otherwise, the agent performs its current trajectory for navigation and updates its memory about the shorted trajectory to reach the goal. This means the number of steps required to reach the goal by this articulated agent under the random walk with memory does not increase over the training procedure.

**Figure 6:**
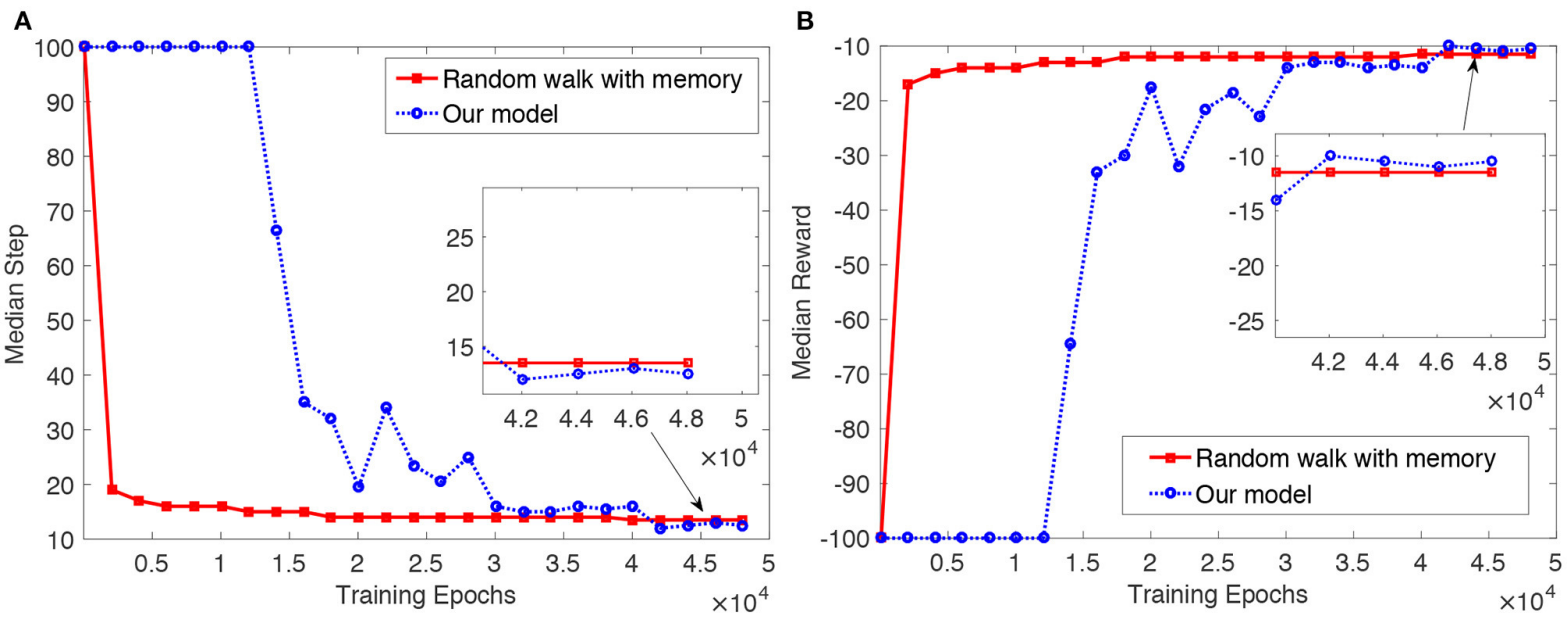
Results of the articulated agent reaching a goal position [surface = 3, x = 1, y = 2] on a 2 × 2 × 2 cube in the random walk with memory and the proposed articulated goal-oriented learning model over 30 tests with different random seeds. For one epoch in every test, if the current exploration trajectory is greater than its remembered shortest trajectory, we let the remembered shortest trajectory be its current exploration trajectory. To smooth the figure, we show the result every 2, 000 epochs. **(A)** Median step per epoch over the 30 tests. **(B)** Median reward per epoch over the 30 tests.

The results are shown in Figure 6. As we can see from Figure 6A, median steps for the random walk with memory and the proposed model decrease as training proceeds. Median steps for the random walk decline rapidly over the first 2,500 epochs, and slowly thereafter. In the proposed model, we see the opposite pattern: median steps fall slowly at first, and then more rapidly. To begin with, the agent in the proposed model randomly explores the object. After the agent finds a more optimal trajectory (i.e., a trajectory with fewer steps) to reach the goal, the actor and critic involved in the proposed model are then trained gradually. Finally, the agent can reach the goal in a more optimal way than the random walk with memory. The median reward over the training epochs shown in Figure 6B also indicates that the articulated agent in the proposed model can reach the goal based on the learned object representation.

Our haptic learning model significantly outperforms the random walk with memory by the end of training. We assess significance using the Mann–Whitney *U*-test, based on median steps from the 4.5 × 10^4^ epoch to the end of the training. (We use nonparametric statistics because the ‘step’ variable isn't normally distributed, due to the cutoff at 100 steps). The one-sided *U*-test finds that over the period (i.e., from the 4.5 × 10^4^ epoch to the end of the training), the median number of steps in the proposed model is significantly smaller than that in the random walk with memory, with the *p* = 3.79 × 10^−316^ < 0.01. By the end of training, the median number of steps for the random walk with memory is 13.5, while for our model it is 12.5—a reduction of 8% of the baseline journey length. (This difference is more clearly appreciated in the inset to Figure 6A). These results demonstrate the utility “to the agent” of the object representation acquired in the proposed tactile exploration model, and complement the “external” evaluations reported in section 4.

**Figure 7:**
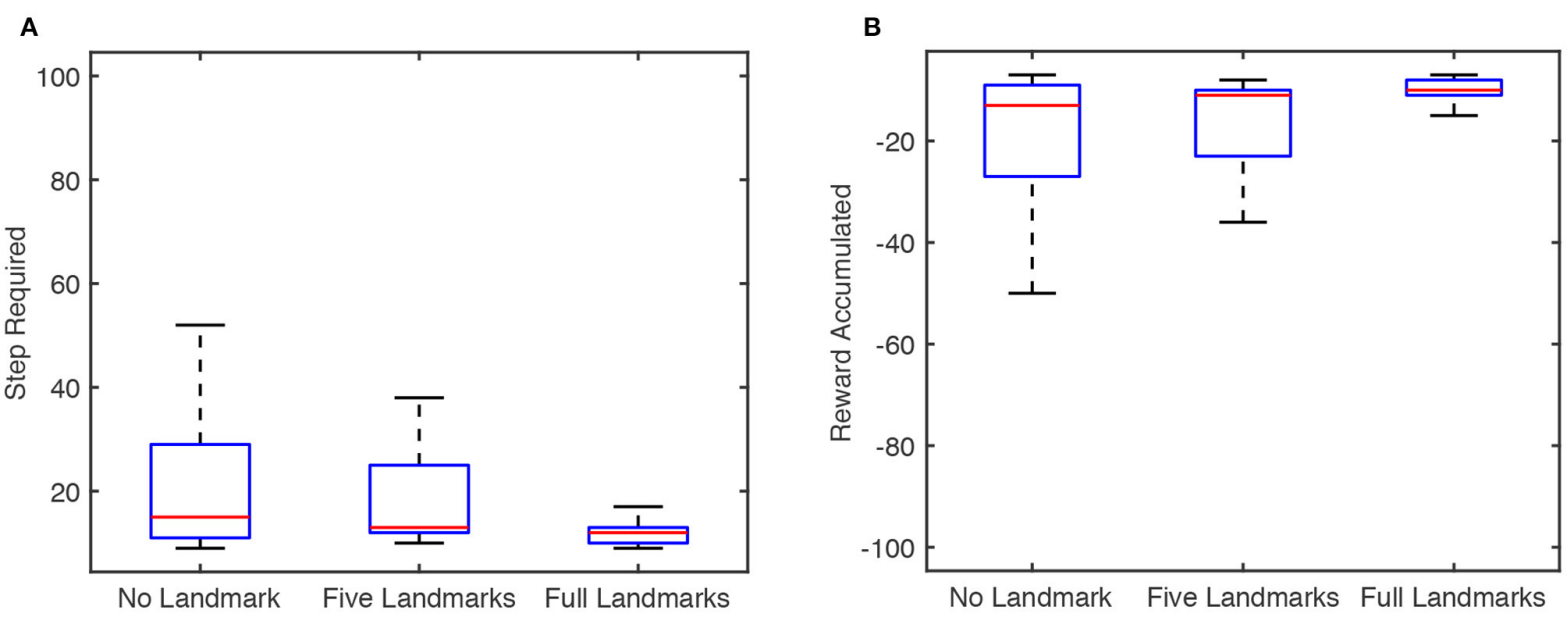
Box plot of results for the articulated agent in the proposed articulated goal-oriented learning model reaching a goal position [surface = 3, x = 1, y = 2] on a 2 × 2 × 2 cube over 30 tests, in which the random seeds are different. The figure is based on the result at the end of each test. The blue box represents the interquartile range (IQR) between the 25% quantile (Q1) and 75% quantile (Q3). The bottom and top edges of the box indicate the Q1 and Q3, respectively. The red horizontal line in the blue box represents the median. The minimum and maximal value in the [*Q*1 − 1.5 ∗ *IQR, Q*3 + 1.5 ∗ *IQR*] are represented as lower and upper black horizontal lines, respectively. **(A)** Step required for the agent to reach the goal on the cube with different numbers of tactile landmarks. **(B)** Reward accumulated for the agent on the cube with different numbers of tactile landmarks.

To further test the proposed model's performance as well as the effects of tactile landmarks, we conduct experiments of the model on the 2 × 2 × 2 cube with different numbers of tactile landmarks. For each number of tactile landmarks, we run the model for 30 tests with random seeds, as before, and we record the number of steps required to reach the goal and the reward accumulated at the end of each test. Results are shown in Figure 7. As shown there, the performance of the reinforcement learning model improves as the number of tactile landmarks increases, both when measured by median step and when measured by median reward.

## 6. Discussion

In this paper, we develop a tactile learning model that learns an implicit representation of the agent's allocentric location on a 3D object being explored. Importantly, this model takes purely ‘egocentric’ stimuli as input—egocentric translation and articulation movements, and egocentric tactile sensations—but it can learn a reasonable approximation of object-centered location. The effectiveness of the model on learning 3D object representations is demonstrated on a cube and cuboid. Comparative results also show the positive effects of object asymmetries, the configuration information of an articulated agent, and tactile landmarks. To examine the potential utility of the acquired knowledge in the articulated tactile learning model, we then propose an articulated goal-oriented learning model. The model takes advantage of the knowledge acquired in MSOM for goal-oriented navigation. Results of the model on a cube and comparisons with a baseline demonstrate the model's effectiveness, and also indicates the utility of the acquired object knowledge in MSOM. The positive effect of tactile landmarks on the model's performance is also suggested.

### 6.1. Remarks on the Scalability of the Proposed Model

The model presented in this paper uses a very simple model of the articulated hand, and is only tested on simple cuboid objects. What are the prospects for scaling it to a more realistic scenario, where a hand with many degrees of freedom explores a natural object, whose geometry includes curves, concavities, and other features?

We see grounds for optimism here. The key finding of the current paper is that a device that learns regularities in sequentially structured inputs provides a useful basis for a model of haptic exploration *by itself*, without any machinery specific to navigation. The model doesn't build in any special knowledge about the properties of objects, or the navigating hand. There are no hard-wired computations performing coordinate transformations, or deriving intermediate representations: it is simply a model of sequence learning. As far as our model is concerned, a more realistic hand, or a more realistic object should just add additional constraints, and additional complexities, to the sequences it is exposed to. If the network learning about these sequences is powerful enough, we expect the representations it learns will be interpretable as information about the hand's object-centered location, as demonstrated here in our simple model.

Naturally, we will need a more powerful model of sequence learning than an MSOM. In particular, we need a model with higher capacity for storing patterns than an MSOM, which is constrained to encode input patterns in relatively localist representations. But such networks are now readily available: for instance, the new generation of Hopfield networks perform the same kind of unsupervised associative learning as an MSOM, but with vastly higher capacity (see e.g., Krotov and Hopfield, 2016, and especially Demircigil et al., 2017). Of course, the question of scalability remains to be addressed empirically—and this is something we plan to pursue in future work. We are particularly interested to see whether an unsupervised sequence-based model can learn to explore objects with curved geometries, rather than the cuboid objects discussed in the current paper.

### 6.2. Extending the Model to Learn Whole Object Representations

Most computational models that learn 3D object representations are based on visual perceptions. The proposed model, on the other hand, is based on touch, and the motor system. The haptic system is what teaches us what objects really are: it directly acquires representations of surfaces and solidity. The visual system can *learn* representations of object geometry—but it must be taught by the haptic system: we feel haptic models can play a useful role in training visual models, as they probably have this role in the human visual system. While the proposed model does not learn perfect representations of allocentric object location, it has the great advantage of parsimony: the MSOM mechanism at its core is a simple model of “clustering” or “chunk-learning,” that encodes frequently-encountered sequences in declarative patterns.

In the current paper, we show that an *articulated* haptic learning model performs better on our evaluation metrics than an unarticulated one. This result tallies with the role of articulation in human haptic exploration: the hand tends to adopt the local shape of the explored object (Klatzky et al., 1990), and thus derives much richer information about the contact surface.

Moving toward an articulated hand model also sets the stage for an account of how the hand can *manipulate* the object being touched. The proposed model provides a useful platform for an account of how the hand can move from being a passive explorer of the surface of an object to an active manipulator, which can move the object, or change its properties.

### 6.3. Future Directions

We could extend the proposed models to investigate the following questions in the future.

- It is obviously important to consider how readily the models are extended to cover objects with curves which don't permit representation using a grid (e.g., spherical balls), and objects with more complex geometries (e.g., cups and chairs). A model extended to cover smooth curves could also inform accounts of how visual representations of curved surfaces are learned—for instance, the inferotemporal representations of curvature discussed in Connor and Knierim (2017). This is a matter of future work.
- Different types of representations of 3D objects can be gleaned from distinct sensorimotor modalities, such as vision and touching. What is the relationship among those representations? How does the brain acquire the linkage? How could we derive a model that learns a function that maps 3D object representations obtained from vision to those acquired in an unsupervised way from haptic exploration? Those questions are a matter of future work.
- Different sized objects can have the same type of shapes. For instance, the 2 × 2 × 2 and 3 × 3 × 3 object has the same shape as a cube. How does the brain project those objects with different dimensions to the same shape category? How do the proposed models offer the ability to learn declarative shape tokens via tactile exploration? Those questions are also a matter for future work.

### 6.4. Some Predictions for Neuroscientists

The model presented here is intended as a model—admittedly a very high-level one—of the brain circuits in humans (and other primates) that control haptic explorations. As mentioned in section 1, we know roughly where in the brain these circuits are to be found: they are in the regions of parietal, premotor, and motor cortex that control the generation of hand/arm movements. But we know very little about the algorithm that is implemented in these circuits. We offer the proposed model as a hypothesis about the nature of this algorithm. We have shown that the model can learn allocentric representations of object location, to some degree of approximation. We also note that the model relies on a simple sequence-learning circuit that is attested elsewhere in the brain (Barone and Joseph, 1989; Elman, 1990).

We finish by making some *predictions* about representations in these areas of the brain that neuroscientists could test. There are several predictions.

- A first prediction is that these brain areas contain cells (or cell assemblies) that respond to specific commonly-occurring sequences of slip sensations. This prediction could be tested using single-cell recordings in monkeys: for instance, using variants on the paradigm employed by Fortier-Poisson and Smith (2016) in primary somatosensory cortex. The same paradigm could be used to investigate higher cortical areas that learn more abstract spatial representations. Good candidates would be parietal areas that have been found to hold object-centered encodings of locations (see e.g., Chafee and Crowe, 2013). In humans, multivoxel decoding techniques are likely to be the most revealing. Again, somatosensory and parietal areas are good targets for investigations, perhaps adapting the paradigm of Kim et al. (2013), which found encodings of vibrotactile stimuli in secondary somatosensory and posterior parietal cortices.
- A second prediction is that these brain areas contain cells (or cell assemblies) that respond to *combinations* of slip sensations and tactile sensations that identify distinctive textures, diagnostic of “tactile landmarks.” (Units of our MSOM have this property). More specifically, we predict that cells (or cell assemblies) respond to specific sequences of slip-sensation, texture and hand-pose combinations—because again, our MSOM units have this property. Similar single-cell and multivoxel-decoding techniques could be used to test these predictions—perhaps extending brain areas to the cuneate nucleus, which is a waystation for hand pose signals and tactile signals (see e.g., Santello et al., 2016).
- If cell assemblies of these kinds are found, we also predict that damage to these assemblies will impair an agent's ability to haptically explore objects. Indicative findings along these lines have already been found for somatosensory cortex: for instance, Hikosaka et al. (1985) showed that muscimol injections in first somatosensory cortex (SI) impaired manipulative behaviors. But our model also predicts that damage in upstream areas (SII, or parietal cortex) will similarly disrupt manipulation.
- We also predict that cells (or cell assemblies) will be found in these brain areas that become active when the agent's hand is at a particular location (and configuration) on a given object, as measured within an object-centered frame of reference. If such cells are found, we also predict these cells will also respond to sequences of slip sensations and/or tactile landmarks, as in the proposed model.
- More directly, we also predict that if the afferent input to upstream brain structures, like the cuneate nucleus as collector of upper limbs electrophysiological signals, is inhibited in some way (e.g., an animal is prevented from obtaining ‘slip’ sensations on the hand by using gloves or local anesthetic), the agent's ability to haptically explore objects and learn their geometries will be impaired.

## Data Availability Statement

The raw data supporting the conclusions of this article will be made available by the authors, without undue reservation.

## Author Contributions

XY and AK drafted the paper. AK and SM revised the paper. All authors contributed to the research described in the article and approved the submitted version.

## Conflict of Interest

The authors declare that the research was conducted in the absence of any commercial or financial relationships that could be construed as a potential conflict of interest.
